# Self-Rated Health and Pain Problems in Mothers of Healthy Children or Children Requiring Outpatient Observation or Hospitalisation: A Pilot Cross-Sectional Study

**DOI:** 10.3390/ijerph18189543

**Published:** 2021-09-10

**Authors:** Anna Aftyka, Wojciech Rosa, Marzena Samardakiewicz

**Affiliations:** 1 Department of Anaesthesiological and Intensive Care Nursing, Faculty of Health Sciences, Medical University of Lublin, 20-093 Lublin, Poland; 2 Department of Applied Mathematics, Faculty of Technology Fundamentals, Lublin University of Technology, 20-618 Lublin, Poland; w.rosa@pollub.pl; 3 Chair and Department of Psychology, Interfaculty Centre for Didactics, Medical University of Lublin, 20-093 Lublin, Poland; marzenasamardakiewicz@umlub.pl

**Keywords:** self-rated health, mothers, distress, depression, anxiety, pain, ICU

## Abstract

A child’s illness or disability is a considerable stressor for the mother and a risk factor for many psychological problems and somatic diseases. The purpose of the study was to (1) assess the prevalence of poor SRH and pain, (2) compare self-rated health and pain, (3) and identify the determinants of SRH and pain in mothers of healthy children and children requiring ambulatory observation or hospitalization. The study covered 234 mothers of both healthy and unhealthy children who required outpatient observation or treatment at an intensive care unit, neonatal intensive care unit, or oncology department. To analyse the variables obtained, the following tools were used: Self-Rated Health, Numerical Rating, Interpersonal Support Evaluation List, Peritraumatic Distress Inventory, Modified Hospital Anxiety and Depression Scale, and Impact of Effects Scale—Revised. The self-assessment of health in mothers of healthy children and those in need of outpatient observation or hospitalization at units with various specialities differed in a statistically significant way. The severity of the average and maximum pain among mothers of healthy children and those with a history of disease differed statistically significantly. Poor SRH co-occurred with severe maximum pain in all of the examined groups. Both in the control group and the group of mothers of children requiring outpatient observation, poor SRH co-occurred with a high level of anxiety. Only in the control group was a correlation found between the severity of the average and maximum pain and the severity of anxiety and depression symptoms.

## 1. Introduction

A child’s illness or disability is a strong stressor for the parents, especially the mother, and a risk factor for many psychological problems and somatic diseases. An existing severe illness or one that occurred in the past have been shown to be associated with an increased incidence of acute stress disorder (ASD), post-traumatic stress disorder (PTSD), increased anxiety, or depression in mothers [1,2,3,4,5,6,7,8,9,10]. Having a child with a disability also has similar consequences. Among other things, the following links have been shown: between the mother’s depression and the presence of cerebral palsy in the child [11]; depression of the mother and Down’s syndrome in the child [12]; depression, anxiety, and suicidal thoughts in the mother and the disability of the child [13]; and PTSD symptoms in the mother and autism of the child [14]. Health problems and the disability of the child are also associated with somatic diseases and chronic pain, with musculoskeletal disorders being the most commonly reported in mothers of children with physical disabilities [15,16]. Authors also report deteriorations in the quality of life associated with physical (somatic) pain in mothers of children with congenital heart defects [17], mothers of children with perinatal shoulder plexus paralysis [18], mothers of children hospitalized for cancer [19], and mothers of children with cerebral palsy [20].

Self-Rated Health (SRH) is a single-item measure, commonly used to assess subjective perception of one’s health, covering a wide range of individual health aspects. It has been demonstrated that this subjective assessment of health is a strong predictor of mortality and morbidity [21,22,23] and the use of hospital treatment [24]. Research to date has shown a relationship between SRH and numerous sociodemographic, psychological, medical, and lifestyle-related variables. Sociodemographic factors in the general population are relatively well understood. A good assessment of one’s health seems to be related to such factors as: being male, young age, being married, employment, high household income, high social class, and high socioeconomic status in comparison with the peer group. It was demonstrated that the effect of the socioeconomic status on SRH can be partially accounted for by the lifestyle and psychological factors [25]. Another factor associated with SRH is ethnicity [26,27], place of residence [28], and the experience of single motherhood [29]. Psychological factors associated with SRH include a satisfying relationship with family and friends [25], individual emotional well-being [30], and self-esteem and optimism [31]. Other studies show that there is a link between SRH and the number of stressful life situations [32] and distress [33]. On the other hand, social support [32] and a sense of self-efficacy are important predictors [33]. Medical factors associated with SRH include depression [25] and multiple morbidity [32]. It has also been demonstrated that a high level of pain is associated with poorer SRH in various groups of subjects [33,34,35]. In addition, Lytzy et al. [26] and Shega et al. [36] demonstrated that chronic pain is linked to poorer SRH. In women, SRH is additionally connected with factors related to the progress of pregnancy and the duration of the last childbirth. It seems that the period of pregnancy causes SRH to become poorer, which slightly improves afterwards, but its initial state is not restored within 12 months of the childbirth [37,38]. Of the factors related to the quality of life, normal body weight and physical activity are significantly linked with SRH [25,32].

Overall, SRH can be taken to refer to a sum of various health aspects which the respondent deems essential, depending on their health conditions, life situation, context, and lifestyle. SRH is mainly accounted for by two components: (1) physical well-being (health) and (2) emotional well-being (health).

The absence of pain is a variable that is tightly connected with physical well-being. Available research suggests that the occurrence of pain in different regions of the body is linked to poor SRH [39]. At the same time, available research results suggest that the illness and disability of the child is related to an increased risk of pain in the mother and worse quality of life related to physical pain [15,16,18,40,41].

In research conducted by Telci et al., pain within the musculoskeletal system in the group of mothers of children with a disability affected almost 80% of the women surveyed and was related to the mobility of the child [16]. A great deal of studies have been conducted on groups of mothers of children with cerebral palsy. In this group of women, Terzi and Tan demonstrated a significantly greater incidence of pain in the musculoskeletal system than in the control group, located most frequently in the lower back. The same research showed that independent risk factors for pain in mothers were as follows: the number of children, the level of functioning of the child with cerebral palsy, the child’s age, and the severity of depression in the mother [15]. A relationship between the functioning of children with cerebral palsy and the severity of pain in mothers was also described by Prudente et al. [40]. Kaya et al. demonstrated a higher intensity of daytime pain and pain connected with movement and a lower quality of life associated with physical pain in mothers of children with cerebral palsy in comparison with mothers of healthy children [41]. Moreover, mothers of children with perinatal brachial plexus paralysis experienced stronger pain than the controls [18]. At the same time there is no research on the incidence and severity of pain in the group of mothers of children with a history of disease but not necessarily connected with the child’s disability. Isolated studies were presented by Sileshi and Terfera and Eyigor et al. The first team showed that mothers of children with heart defects reported a lower quality of life in the context of physical pain than mothers of healthy children, but no significant differences between mothers of children with severe heart defects and mothers of children with mild to moderate severe heart defects were noted [17]. Interestingly, Eyigor et al. did not observe any statistically significant differences in the quality of life between mothers of children with cancer and mothers of healthy children in terms of quality of life related to physical pain, although the quality of life related to both physical and mental health in mothers of ill children was significantly lower [19]. Most studies on pain in mothers of children with a disability focus on musculoskeletal pain or back pain, others are related to quality of life related to physical pain. Some research interprets the presence of pain in terms of a dichotomous variable, which may not be sufficient from the clinical perspective. We have not found any study on the relationship between SRH and pain intensity. We believe that the gap in this research area is worth filling.

Disturbed emotional well-being (health) in the group of mothers of both healthy and ill children is linked, in light of scientific research, to the occurrence of depressive symptoms, increased anxiety, acute stress disorder, and posttraumatic stress disorder, as well as peritraumatic distress [1,2,3,4,5,6,7,8,9,10]. While the occurrence of ASD and PTSD symptoms is reported more often in the group of parents with children treated at NICUs, PICUs, and pediatric oncology units [1,2,3,4,6,8], depression and anxiety are frequently a significant problem in parents of children with a chronic disease and those with a disability [7,9,10,11,12]. Although the co-occurrence of poor SRH and symptoms of depression and anxiety is relatively well described in the literature, the number of studies exploring the association of acute stress disorder with posttraumatic stress disorder (PTSD) is limited.

Summing up, we can say that, so far, publications dealing with the incidence of pain and poor self-assessment of health and the severity of pain in mothers of children experiencing various types of health problems are rather few. The relationship between SRH and pain intensity and sociodemographic, psychological, and medical variables is also little understood. Therefore, it seems appropriate to conduct research on the occurrence of SRH and pain in mothers of healthy children and mothers of children in need of ambulatory observation due to eventful perinatal history or hospitalization.

## 2. Materials and Methods

The purpose of the study was to: (1) assess the prevalence of poor SRH and pain, (2) compare self-rated health and pain, and (3) identify the determinants of self-assessment of health and pain in both mothers of healthy children and those of children requiring ambulatory observation or hospitalization. The last of our research goals envisaged an assessment of the co-occurrence of a weak SRH and pain problems (average and maximum), as well as symptoms of anxiety, depression, peritraumatic distress, PTSD, and selected sociodemographic variables in individual studied groups.

### 2.1. Study Design

The study covered mothers of healthy children and those requiring ambulatory observation or hospitalization. Mothers of children requiring hospitalization were interviewed while their children were in hospital care. After they were informed of the aim and procedure of the study, the mothers were asked to give their written informed consent to the survey. Then, they were handed the questionnaire placed in an addressed envelope, together with instructions how to return the questionnaire. The study of the mothers of healthy children was conducted with the use of targeted selection so that their sociodemographic data (age, place of residence, education) were similar to the sociodemographic data of mothers of sick children. However, this was hard because of difficulty obtaining informed consent from mothers living in rural areas and mothers with the lowest education. The STROBE guidelines were applied to ensure high quality research results [42].

### 2.2. Setting

The study was conducted from 1 September 2016 to 31 December 2017. Data concerning mothers of children requiring outpatient observation or hospitalization at a NICU or PICU were acquired in the only clinical children’s hospital located in Lublin Voivodeship, in the northeastern part of Poland (Central Europe). Data on mothers of healthy children were also acquired in this voivodeship.

### 2.3. Participants

The study covered 234 mothers including 61 mothers of healthy children (Control Group) and 173 mothers of children requiring outpatient observation or hospitalization. The response rate was 70.91%. Another group included mothers of children in need of observation at a rehabilitation clinic due to an eventful perinatal history and mothers of children treated at the Neonatal Intensive Care Unit (NICU), Paediatric Intensive Care Unit (PICU), and the Department of Paediatric Oncology (DPO). Correctly completed questionnaires were returned by 48 mothers of newborns treated at the NICU, 33 mothers of children treated at the PICU, 61 mothers of paediatric patients treated at the DPO, 31 mothers of children requiring observation at the rehabilitation clinic, and 61 mothers of healthy children. The inclusion criteria were: reaching the age of 18, knowledge of the Polish language in speech and writing, and the written consent to participate in the study. In the case of mothers of NICU and PICU patients, those whose children had been hospitalised at an ICU for at least 3 days were qualified for the study. In the case of mothers of children treated for cancer, the study included those whose children had been diagnosed in the previous 6 months.

### 2.4. Research Tools

The study employed a number of standardised research tools:

Self-Rated Health (SRH). SRH is a measure frequently used in epidemiological studies to assess the health of a population. In this study, similarly to health self-assessment surveys conducted by the Polish Central Statistical Office (GUS), the respondents were asked not to consider short-term or temporary health problems (e.g., colds, flu) when answering the question “How do you rate your health?” The respondents had a choice of 5 answers: 1 = very good, 2 = good, 3 = so-so, neither good nor poor, 4 = poor, 5 = very poor. SRH is commonly used in many countries and cultures [43,44,45,46] as well as different age groups [47,48,49]. Here, a dichotomisation of SRH was possible, and so it was also performed in this study. Under this scheme, the respondents are divided into those who rate their health either as “very poor”, “poor”, or “moderate” (poor SRH) and those who rate their health as “very good” or “good” (good SRH) [50].

The Numerical Rating Scale (NRS) was used to assess pain symptoms. The 11-degree scale was used, where 0 stands for a total absence of pain, while 10 denotes unbearable pain (the worst imaginable pain). The NRS was employed to determine the average and maximum severity of pain felt in the last week.

The Interpersonal Support Evaluation List (ISEL-40 v. GP) was used to assess perceived social support. In its general population version, the survey contains 40 dichotomous statements with two possible answers: “probably true” or “probably false”. It is designed to estimate the potential possibility of gaining social support as envisaged by the respondents. The original version of ISEL 40 v. GP has 4 10-item subscales: tangible support, appraisal support, self-esteem support, and belonging support [51,52,53]. Due to the nature of the research and the low internal consistency of the subscales in the study group, it was decided to use only the total score. ISEL-40 v. GP internal consistency, expressed with Cronbach’s α coefficient, was 0.91. In the evaluation of the stability of results with the use of the test–retest method, satisfactory reliability results were obtained, too (α = 0.92) [52]. In our research group, ISEL-40 v. GP showed strong internal consistency, with a Cronbach alpha of 0.81.

Peritraumatic Distress Inventory (PDI) was used to assess the level of stress during or immediately after traumatic events. Peritraumatic distress is defined as the emotional and physiological distress experienced during and/or immediately after a traumatic event and is associated with the development and severity of posttraumatic stress disorder (PTSD) and related psychological difficulties [54]. The Polish version of the inventory used for mothers of healthy children and children with an eventful perinatal history consists of 11 statements, which make up 2 factors: (1) Negative emotions, (2) Sense of threat and somatic reactions [55]. Cronbach’s α coefficient for the entire scale, in the validation group of employees of the Polish emergency medical system, was 0.77. In this group, there was a strong positive correlation between peritraumatic distress and severity of posttraumatic stress symptoms, which confirms the validity of the tool [55]. In our research group, PDI showed strong internal consistency, with a Cronbach’s α of 0.83 for the total scale, 0.87 for the Negative Emotions subscales, and 0.80 for the Sense of Threat and Somatic Reactions subscales.

The mothers of healthy children with an eventful perinatal history and mothers of children treated at the oncology department were given additional questionnaires:

Hospital Anxiety and Depression Scale Modified (HADS-M) was used to assess the severity of symptoms of depression, anxiety, and anger. The HADS-M is a self-report scale containing 16 statements, 7 of which are related to anxiety, 7 to depression, and 2 to the expression of anger. The anxiety and depressive subscales are also valid measures of severity of the emotional disorder [56]. The Polish version was found to be the tool that has satisfactory psychometric properties. HADS-M internal consistency, expressed with Cronbach’s α coefficient, was 0.80 for the Anxiety scale, 0.85 for Depression scale, and 0.76 for Anger expression scale. The accuracy of HADS-M was estimated by the correlation of its results with the EORTC QLQ-C30 performance scales [56]. In the group of patients with psychosomatic diseases, the level of depression assessed with the use of HADS was strongly positively correlated with the severity of depression in the Beck Depression Inventory and the level of anxiety assessed with the use of HADS—with the severity of anxiety as a trait in the State-Trait Anxiety Inventory [57]. Sensitivity, specificity, PPV, and NPV of the HADS and HADS-M in the general population in Poland are unknown. In the group of patients after ischaemic schock, using the cut-off value ≥7 points, the results were as follows: depression subscale: sensitivity 90.0%, specificity 92.2%; anxiety subscale: sensitivity 86.5%, specificity 94.9%, which was the most optimal cut-off point [58]. In our research group, Cronbach’s alpha was 0.90 for the total scale, 0.87 for the Anxiety subscale, and 0.90 for the Depression subscale.

Impact of Event Scale—Revised (IES-R) was used to assess subjective stress caused by a traumatic event. The tool consists of 22 statements and contains 3 subscales: Intrusion, Arousal, and Avoidance, which are relevant for the symptoms of post-traumatic stress disorder (PTSD) [59,60]. The Polish version of IES-R was found to be the tool that has satisfactory psychometric properties. IES-R internal consistency, expressed with Cronbach’s α coefficient, is 0.92 for the entire scale, and for Intrusion, Arousal, and Avoidance, it is 0.89, 0.85, and 0.78, respectively. Stability of the IES-R results with the use of the test–retest method for the entire scale is 0.75, and for Intrusion, Arousal, and Avoidance, it is 0.79, 0.76, and 0.68, respectively [60]. Sensitivity, specificity, PPV, and NPV of the IES-R in the general population in Poland are unknown.

The use of these tools on a limited group of respondents was due to: (1) the short average duration of treatment at the NICU and (2) the diagnostic criteria for depression and PTSD related to the duration of symptoms (2 and 4 weeks, respectively).

### 2.5. Ethical Approval

The research project gained approval of the Bioethics Committee of the Medical University of Lublin (KE-0254/119/2015).

### 2.6. Statistical Analyses

For statistical analysis, the R 3.4.2 environment was used (Short Summer 28 September 2017). While planning the research, our assumption was to check if there were any differences in perceived pain and self-rated health in the studied groups, compare the distribution of the selected variables, and examine the correlation between the studied quantitative variables. The adopted significance level was *p* < 0.05. The normality of distribution was tested by means of the Shapiro–Wilk test. The tested variables differed significantly from the standard normal distribution so the non-parametric Kruskal–Wallis test was used for comparison. Correlations between the categories were analysed using Yates’s chi-squared test, corrected for continuity. Correlations between the scales were studied using Spearman’s rho.

## 3. Results

### 3.1. Descriptive Data

The mean age of the mothers was 34.27 ± 6.32 years. The respondents from particular departments differed with a statistical significance in terms of most sociodemographic features, such as age, education, place of residence, and occupation. The study groups also differed in terms of the presence of chronic diseases in their medical history, the assessment of the sick child’s health condition, and the presence of a disability. These differences are related to two issues: (1) the specificity of patients in individual departments and their mothers and (2) the different percentages of returned questionnaires in individual groups. The first situation accounts for, among others, differences in the age of mothers and the youngest child, mothers’ professional activity, the child’s health, and the presence of disability in the child, and the other situation explains differences in education and place of residence. Detailed characteristics of the examined mothers are presented in Table 1.

### 3.2. Main Results

A number of statistically significant differences in the biopsychosocial well-being of the mothers of healthy children and children requiring ambulatory observation or hospitalization were found. The mothers of healthy children, more often than the others, manifested very good or good health (*n* = 53; 86.89%) and a lower severity of average (NRS = 2.07 ± 1.67) and maximum (NRS = 3.19 ± 2.00) pain felt in the last week. There were also statistically significant differences in peri-traumatic distress, negative emotions, sense of threat, depression, anxiety, and PTSD and its symptoms between the mothers of healthy children and the mothers of children requiring ambulatory observation or hospitalization. Generally speaking, the mothers of healthy children are characterized by the lowest intensity of these variables, the mothers of children requiring outpatient observation show slightly higher intensity, and the mothers of children requiring hospitalization show the highest. Detailed results of the comparisons are presented in Table 2.

Significant differences in self-rated health were found in the mothers belonging to individual groups (χ^2^ = 29.643; *p* = 0.003). The respondents most often described their health condition as good—about half of the surveyed women from each group gave this assessment. Almost one third of the control group (*n* = 19; 31.67%) and only 1 in 30 respondents whose children were treated at the oncology department (*n* = 2; 3.28%) assessed their health as very good. The “so-so” assessment regarding health is relatively common in the group of mothers of children with health problems, and its frequency is by far the highest in the group of mothers of children treated for cancer (*n* = 26; 42.62%). Only several women assessed their health as poor, and those were only the mothers of children with health problems: children treated at the NICU (*n* = 3; 6.25%), at the oncology department (*n* = 3; 4.92%), and children with an eventful perinatal history (*n* = 1; 3.23%). These results suggest that the postnatal period and the oncological disease of a child may be associated with a reduced self-assessment of health. A detailed comparison of the groups distinguished according to the place of hospitalization of the child with respect to self-rated health status is presented in Table 3.

Significant differences were found in the intensity of average pain experienced by the respondents in the last seven days as per individual groups (χ^2^ = 19.59; *p* = 0.012). Generally speaking, women who felt no or minor pain predominated. In the control group, their proportion was 86.2%, and it was 77.4% in the group of mothers of children with an eventful perinatal history. The proportions of mothers of children treated at the department of oncology, the ICU, and NICU were 68.9%, 67.7%, and 47.8%, respectively. It is worth noting that moderate or strong pain was experienced by more than half of the mothers whose children were treated at the NICU, one in three women whose child was treated at the oncology department or ICU, and one in four respondents with a child with a medical history. A detailed comparison of the groups distinguished by the place of hospitalization of the child with respect to the severity of average pain is presented in Table 4.

There were also significant differences in the intensity of maximum pain perceived by the respondents during the last seven days as per individual groups (χ^2^ = 32.78; *p* < 0.001). These differences were even greater than in the case of the intensity of average pain. Overall, about every second woman in each of the tested groups described the strongest pain felt in the last seven days as not exceeding 3 points on the NRS scale. The absence of pain or pain with intensity of less than 3 points was reported by 55.2% of the mothers in the control group, 48.4% of the mothers of children treated at the ICU, 52.5% of the mothers of children treated at the oncology department, and 61.3% of those of children with an eventful perinatal history. At the same time, only 28.9% of the mothers of children treated at the ICU described their most intense pain as mild. Strong pain was felt by more than a third of the women whose child required treatment at the NICU and by 1 in 10 mothers whose child was treated at the oncology department, the ICU, or had an eventful perinatal history. At the same time, severe pain was very rarely reported in the mothers in the control group, but relatively often, the presence of moderate pain was reported. A detailed comparison of the groups distinguished by the place of hospitalization of the child with respect to the severity of average pain is presented in Table 5.

A number of correlations between SRH and demographic, medical, and psychological variables were found. In all groups of mothers, a statistically significant relationship or one at the limit of statistical significance was found between SRH (with high values for poor health) and the severity of the average and maximum pain experienced within the last seven days. In all these groups, poor health was associated with a high severity of average and maximum pain. The mothers of children treated at the NICU manifested a positive correlation between SRH and peritraumatic distress (rho = 0.47, *p* < 0.01) and its dimensions, i.e., negative emotions (rho = 0.36, *p* < 0.05) and a sense of threat (rho = 0.48, *p* < 0.01). In this group of women, a negative correlation between SRH and social support was also found (rho = –0.47, *p* < 0.01). This means that high SRH scores, indicating poor health, co-occur with low social support. A similar correlation at the limit of statistical significance concerns the mothers whose children are treated at the oncology ward. A positive correlation between SRH and the age of the youngest child was found in the control group and in the oncology department (rho = 0.36, *p* < 0.05 and rho = 0.49, *p* < 0.001, respectively). This means that high SRH scores, indicating poor health, co-occur in these groups of mothers with the older age of the youngest child. Interestingly, the correlations between SRH and mother’s age are not statistically significant. Positive correlations between SRH and anxiety were also found in the control group (rho = 0.31, *p* < 0.05), in the mothers of children treated in the oncology department (rho = 0.27, *p* < 0.05), and in the mothers of children with an eventful perinatal history (rho = 0.39, *p* < 0.05). In the latter group, a significant positive correlation was also found between SRH and the severity of depressive symptoms (rho = 0.44, *p* < 0.05). In the mothers of children treated at the oncology department, SRH was positively correlated with the severity of arousal symptoms (rho = 0.28, *p* < 0.05). The correlation matrix is shown in Table 6.

A positive correlation was found between the mother’s BMI and the intensity of average pain in the group of mothers of children treated at the NICU (rho = 0.34, *p* < 0.05). A positive correlation between the level of average pain and the level of anxiety (rho = 0.45; *p* < 0.001) and depression (rho = 0.37; *p* < 0.01) was observed in the control group; a slightly similar correlation, although statistically insignificant, was observed in the group of mothers whose children had an eventful perinatal history. In the latter group, a negative correlation between the level of average pain and the severity of anger was demonstrated (rho = –0.41, *p* < 0.05) and avoidance symptoms (rho = –0.44, *p* < 0.05). The matrix of correlations between the variables is shown in Table 7.

As regards the maximum pain experienced, similar correlations were found as in the case of average pain intensity: a positive correlation was found between the mother’s BMI and maximum pain (rho = 0.37, *p* < 0.05) and a negative correlation between the age of the youngest child and the intensity of maximum pain felt (rho = –0.41, *p* < 0.05) in the group of mothers of children treated at the NICU. There was also a negative correlation (at the limit of statistical significance) between the level of maximum pain and social support received by the mothers of children treated at the NICU and ICU. A positive correlation was observed between the level of maximum pain and the level of anxiety (rho = 0.40; *p* < 0.01) and depression (rho = 0.37; *p* < 0.01) in the control group. A similar correlation, though statistically insignificant, was observed in the group of mothers whose children had an eventful perinatal history. A negative correlation between the intensity of maximum pain and anger was noticed in the group of mothers of children with an eventful perinatal history (rho = –0.53, *p* < 0.001) and the control group (rho = –0.31; *p* < 0.05). The intensity of maximum pain also correlated with the level of avoidance in the group of mothers of children with an eventful perinatal history (rho = –0.44, *p* < 0.001). The matrix of correlations between the variables is shown in Table 8.

## 4. Discussion

Over the last 5 years, Poles’ subjective assessment of their health has slightly improved. While in the year 2009, over 34% of Polish respondents of a representative sample assessed their health as below good (i.e., so-so, poor, or very poor), at the end of 2014, such opinions were shown by fewer than 33% of the Polish population [61]. In this context, our results for mothers of children with a medical history with respect to SRH are alarming—even relatively young women presented scores comparable to those of the ageing Polish population. In the general population of Polish women, poor SRH (very poor, poor, or so-so, neither good nor bad) was found in 9.9% of the respondents aged 20 to 29 and 16.2% of the women aged 30 to 39 [62]. In this study, 13.33% of the controls, where the average age was almost 35, described their health as “so-so, neither good nor bad”, but no subject in this group described her health as “poor” or “very poor.” The results obtained in the control group are similar to those obtained for a representative sample of Polish women, whereas in all groups of mothers of children with a disease history the incidence of poor SRH is higher.

Our results indicate that the self-assessment of health in the specific groups differed statistically significantly. The lowest percentage of respondents describing their health condition as good or very good was observed among the mothers of children with cancer. Only every other respondent from this group assessed her health as good or very good. These results are consistent with those obtained by Eyigor et al., where mothers of children with cancer presented significantly lower scores than the controls in terms of quality of life related to general health, vitality, and mental health [19]. Slightly better results, but also low, were obtained by mothers of children treated at the NICU and mothers of children with an eventful perinatal history. Both groups are characterised by a relatively short time span since the last birth, and this factor is regarded by the literature as related to a reduced SHR [37,38]. Generally speaking, mothers of newborns and infants treated at a NICU or requiring observation due to perinatal history tended to show a negative correlation between SRH and the age of the youngest child. In mothers of children in the control group and in those of children with cancer, the opposite situation was observed since a significant correlation between the age of the youngest child and the mother’s SRH was found. At the same time, no statistically significant relationship between SRH and the mother’s age was found in any of the studied groups, though a tendency to show worse SRH was noticed in the older mothers in several groups, which is consistent with other reports to date [63].

The group of mothers whose children were treated at the NICU and those whose children were monitored owing to their perinatal history were linked in terms of the intensity of peritraumatic distress and its dimensions and SRH. This relationship was statistically significant in the group of mothers of children treated in the NICU, but not in the other, a fact which can be associated with a small number of respondents involved. Additionally, peritraumatic distress in the group of mothers treated at the NICU was the greatest. So far, this relationship has not been described in the literature, but there are reports of a relationship between stress and SRH [63].

The presented research demonstrates the co-occurrence of average and maximum pain symptoms and weak SRH both in mothers of both ill and healthy children. This predictor proved to be, unlike many others, universal for all of the studied groups, though in some—probably owing to the small number of respondents in them—correlations existed at the limit of statistical significance. Generally speaking, weak SRH was more strongly associated with the severity of maximum pain than with the intensity of average pain. Similar results were obtained in the examinations of mothers of newborns whose physical problems such as fatigue and musculoskeletal and abdominal pains increased the risk of poor SRH both in primiparous and multiparous [64]. The association between back pains and SRH was found in a group of Swedish women with an advanced pregnancy, but the relationship was not observed in those respondents one year after birth [65].

In Poland, 57% of women in the general female population experienced physical pain in the last 4 weeks. Obviously, the perception of pain increased with age. Pain problems affected 35% of women aged 20–29, 42% of those aged 30–39, and 54% aged 50–59 [61].

In the literature of the subject, the intensity of pain in mothers of both healthy children and those with a history of disease was rarely reported for direct measurements. Quality of life is studied more often and popular tools such as the SF-36 include questions about pain. Our study showed that the severity of both average and maximum pain varied between the groups and was the lowest in the group of mothers of healthy children. This is consistent with the results observed in the majority of available studies [15,16,17,18,20,41]. An interesting study on pain in mothers of children with cerebral palsy was conducted by Kaya et al. Similarly to ours, mothers were asked about the perceived intensity of pain during the day, at night and when moving around in the last week using the Visual Analogue Scale (VAS). Mothers of children with cerebral palsy felt significantly stronger pain during the day and while moving around than the controls, whereas the intensity of pain at night did not differ significantly statistically. In addition, the study based on the SF-36, specifically its Bodily Pain subscale, showed significant differences between the groups [41]. Eyigor obtained results that were different from those described in the literature and ours while examining mothers of children with cancer. This group did not differ from the control group in terms of results obtained in the frequently used Bodily Pain scale, while scoring significantly lower for quality of life related to general health, vitality, and mental health [19].

By definition, pain is a receptor and emotional sensation, therefore it was considered desirable to examine correlations between the intensity of average and maximum pain and the level of anxiety and depression. The results confirmed the existence of statistically significant correlations only in the control group. This can be partly explained by the small number of respondents, but the values of the correlation coefficient linking the intensity of average pain and the level of anxiety and depression in the group of mothers of children with cancer are puzzling, being close to zero. Combining this result with the highest percentage of poor SHR in the examined group may suggest a higher incidence of somatic and/or psychosomatic diseases in this group of subjects. The downside of the proposed explanation of this state of affairs is the low incidence of chronic diseases in the group of mothers of children with cancer in comparison with the control group and the other studied groups of mothers. For this reason, further research on this issue seems fully justified.

The presented study showed a negative correlation between anger, or rather, its expression, and the severity of average and maximal pain in mothers of healthy children and mothers of children requiring outpatient observation. Relationships between these variables are typically studied and discussed with regard to groups of people with chronic pain [65,66,67]. Interesting results were presented by Kerns et al., who showed that the style of suppressing angry feelings was the strongest predictor for reported pain intensity and pain behaviours in the group of variables, including demographics, pain history, depression, anger intensity, and other anger expression styles [66]. The significance of the confirmation of this phenomenon in the studied group is not yet clear. It seems advisable to carry out experimental studies assessing the effectiveness of interventions based on the teaching of constructive methods of anger expression with respect to the intensity of pain in women in severe pain.

Limitation. The limitations of the study include the small size of the studied groups and differing sociodemographic variables among the groups. These restrictions, however, are typical of pilot studies.

Interpretation. The presented results suggest that the subject area related to the severity of pain and health self-assessment in mothers of healthy children and those with an eventful perinatal history should be explored in scientific research. The research conducted in the group of mothers of newborns and infants and seriously ill children, including those treated at intensive care units, neonatal intensive care units, or oncology departments seem to have particular importance.

Generalisability. For now, the results should not be generalized, and we recommend further research in this subject.

## 5. Conclusions

Self-rated health in mothers of healthy children and those in need of outpatient observation or hospitalization at units with various specialities differs in a statistically significant way. The severity of average and maximum pain in mothers of healthy children, those with eventful perinatal history, and those requiring hospitalization at units with various specialities differs in a statistically significant way. Poor SRH coexists with a high intensity of pain in mothers of both healthy children and those with an eventful perinatal history or that are hospitalized at units with various specialities. Both in the control group and in the group of mothers of children with eventful perinatal history, poor SRH co-occurs with a high severity of anxiety. This relationship was not observed in any of the groups of mothers of hospitalized children. Only in the control group was a correlation found between the severity of average and maximum pain and the severity of anxiety and depression symptoms. A similar relationship was not observed in any of the groups of mothers of children with health problems.

These conclusions are very general, which undoubtedly puts a limitation on this study. However, it shows a wider context of the problem. Mothers in four of the five studied groups experienced the sickness of their children, but their pain problems and SRH varied. Those groups differed also in terms of the incidence and correlation between pain problems and SRH and selected sociodemographic and psychological variables. These data are important and should be accounted for when planning interventions intended to increase the well-being of mothers of children in need of treatment in different hospital wards. It seems that despite certain similarities, the studied groups require preventive interventions that fit their specific profiles. From the clinical perspective, it seems important to implement screening for pain problems in mothers of children treated in hospital, especially in NICUs. It is also advisable to extend research, especially longitudinal and interventionist research, on pain and low SRH in mothers of children with various health records and mothers of healthy children.

## Figures and Tables

**Table 1:** The characteristics of the examined group.

		Controls (*N* = 61)	ICU (*N* = 33)	NICU (*N* = 48)	Oncology (*N* = 61)	Observation (*N* = 31)	Test	*p* Value
Age, years (20–53)		34.85 (5.55)	33.12 (6.89)	31.02 (5.44)	36.82 (6.90)	34.40 (4.80)	H = 23.52	*p* < 0.001
Age of youngest child, years (0–17)		4.68 (4.49)	3.94 (4.99)	0.07 (0.11)	6.25 (5.02)	0.46 (0.30)	H = 114.31	*p* < 0.001
Education *: *n* (%)	Vocational	1 (1.64)	11 (33.33)	3 (6.25%)	8 (13.11)	4 (12.90)	χ^2^ = 32.11	*p* < 0.001
	Secondary or postsecondary	14 (22.95)	8 (24.24)	12 (25.00)	23 (37.70)	4 (12.90)		
	Higher	46 (75.41)	14 (42.42)	33 (68.75)	30 (49.18)	23 (74.19)		
Residence: city, *n* (%)		48 (78.69)	13 (39.39)	27 (56.25)	38 (62.30)	18 (58.06)	χ^2^ = 15.16	*p* = 0.004
Marital status: married, *n* (%)		51 (83.61)	30 (90.91)	40 (83.33)	51 (83.61)	29 (93.55)	χ^2^ = 2.97	*p* = 0.563
Financial situation, *n* (%)	Very good	7 (11.48)	4 (12.12)	8 (16.67)	5 (8.20)	3 (9.68)	χ^2^ = 12.34	*p* = 0.137
	Good	47 (77.05)	20 (60.61)	30 (62.50)	34 (55.74)	21 (67.74)		
	Modest	7 (11.48)	9 (27.27)	10 (20.83)	22 (36.07)	7 (22.58)		
Employment, *n* (%)		51 (83.61)	21 (63.64)	34 (70.83)	34 (55.74)	22 (70.97)	χ^2^ = 11.72	*p* = 0.020
Partner’s employment, *n* (%)		54 (88.52)	30 (90.91)	39 (81.25)	51 (83.61)	30 (96.77)	χ^2^ = 5.27	*p* = 0.261
Chronic diseases, *n* (%)		11 (18.03)	5 (15.15)	18 (37.50)	9 (14.75)	8 (25.81)	χ^2^ = 10.37	*p* = 0.035
Partner’s chronic diseases, *n* (%)		10 (16.39)	4 (12.12)	6 (12.50)	5 (8.20)	6 (19.35)	χ^2^ = 2.95	*p* = 0.567
Child’s health: very good or good, *n* (%)		57 (93.44)	14 (42.42)	12 (25.00)	24 (39.34)	23 (74.19)	χ^2^ = 66.78	*p* < 0.001
Child’s disability: current, *n* (%)		0 (0.00)	13 (39.39)	11 (22.92)	20 (32.79)	6 (19.35)	χ^2^ = 27.84	*p* < 0.001

H—Kruskal-Wallis test, χ^2^—Chi^2^ test, * Education: Vocational (usually 13 schooling years), Secondary or postsecondary (usually 13–16 schooling years, often with A-level certificate), Higher (usually 16–18 schooling years, bachelor’s or master’s degree).

**Table 2:** A comparison of groups distinguished by the place of hospitalization of the child with respect to selected biopsychosocial welfare features of mothers of healthy children and those requiring outpatient observation or hospitalization.

	Controls	ICU	NICU	Oncology	Observation	Test	*p* Value
SRH: very good or good, *n* (%)	53 (86.89)	26 (78.79)	34 (70.83)	32 (52.46)	22 (70.97)	χ^2^ = 18.76	*p* < 0.001
Average pain (0–10)	2.07 (1.67)	3.03 (2.39)	3.61 (2.25)	2.66 (2.21)	2.35 (2.11)	H = 13.83	*p* = 0.008
Maximum pain (0–10)	3.19 (2.00)	3.58 (2.42)	5.31 (3.27)	3.36 (2.56)	2.90 (2.44)	H = 15.13	*p* = 0.004
Peritraumatic distress (0–44)	14.30 (7.57)	23.03 (9.65)	24.66 (7.26)	23.19 (7.90)	18.32 (6.52)	H = 45.91	*p* < 0.001
Negative emotions (0–20)	10.12 (4.76)	14.83 (4.65)	15.78 (3.30)	14.97 (3.84)	13.04 (4.26)	H = 45.61	*p* < 0.001
Sense of threat (0–24)	4.18 (3.81)	8.21 (6.44)	8.88 (5.07)	8.22 (5.56)	5.29 (3.81)	H = 29.10	*p* < 0.001
Depression (7–28)	10.86 (3.21)	X	X	16.83 (4.81)	13.10 (4.18)	H = 43.04	*p* < 0.001
Anxiety (7–27)	12.57 (3.47)	X	X	18.66 (4.75)	13.73 (4.23)	H = 44.83	*p* < 0.001
PTSD (0–4)	1.56 (0.84)	X	X	2.42 (0.78)	1.73 (0.68)	H = 26.45	*p* < 0.001
Intrusion (0–4)	1.58 (0.99)	X	X	2.66 (0.84)	2.13 (0.94)	H = 30.99	*p* < 0.001
Arousal (0–4)	1.63 (0.88)	X	X	2.51 (0.82)	1.59 (0.87)	H = 28.36	*p* < 0.001
Avoidance (0–4)	1.45 (0.94)	X	X	2.05 (0.95)	1.37 (0.63)	H = 12.79	*p* = 0.002

H—Kruskal-Wallis test, χ^2^—Chi^2^ test.

**Table 3:** A comparison of the groups distinguished according to the place of hospitalization of the child with respect to self-rated health.

SRH Level	Controls	ICU	NICU	Oncology	Observation	Test	*p* Value
Very good	19 (31.67%)	8 (24.24%)	11 (22.92%)	2 (3.28%)	6 (19.35%)	χ^2^ = 29.643	*p* = 0.003
Good	33 (55.00%)	18 (54.55%)	23 (47.92%)	30 (49.18%)	16 (51.61%)		
So-so	8 (13.33%)	7 (21.21%)	11 (22.92%)	26 (42.62%)	8 (25.81%)		
Poor	0 (0.00%)	0 (0.00%)	3 (6.25%)	3 (4.92%)	1 (3.23%)		

χ^2^—Chi^2^ test.

**Table 4:** A comparison of groups distinguished according to the place of hospitalization of the child with respect to the severity of average pain.

Level of Pain	Control	ICU	NICU	Oncology	Observation	Test	*p* Value
No or mild (0–3)	50 (86.21%)	21 (67.74%)	22 (47.83%)	42 (68.85%)	24 (77.42%)	χ^2^ = 19.59	*p* = 0.012
Moderate (4–6)	7 (12.07%)	8 (25.81%)	20 (43.48%)	15 (24.59%)	5 (16.13%)		
Severe (7–10)	1 (1.72%)	2 (6.45%)	4 (8.70%)	4 (6.56%)	2 (6.45%)		

χ^2^—Chi^2^ test.

**Table 5:** A comparison of groups distinguished according to the place of hospitalization of the child with respect to the severity of maximum pain.

Level of Pain	Controls	ICU	NICU	Oncology	Observation	Test	*p* Value
No or mild (0–3)	32 (55.17%)	15 (48.39%)	13 (28.89%)	32 (52.46%)	19 (61.29%)	χ^2^ = 32.78	*p* < 0.001
Moderate (4–6)	25 (43.10%)	13 (41.94%)	15 (33.33%)	22 (36.07%)	9 (29.03%)		
Severe (7–10)	1 (1.72%)	3 (9.68%)	17 (37.78%)	7 (11.48%)	3 (9.68%)		

χ^2^—Chi^2^ test.

**Table 6:** Correlations between SRH and selected demographic, medical, and psychological variables (Spearman’s rho).

	Controls	ICU	NICU	Oncology	Observation
Age	0.17	−0.01	0.05	0.21	0.15
Age of youngest child	0.36 *	0.17	−0.15	0.49 ***^,##^	−0.13
BMI	0.17	−0.19	0.20	−0.01	−0.32
No. of pregnancies	0.07	−0.23	0.16	−0.03	−0.21
No. of births	0.05	−0.19	0.19	−0.01	−0.29
Social support	−0.15	−0.09	−0.47 **^,#^	−0.27	−0.08
Average pain	0.46 ***^,##^	0.37 *	0.29	0.24.	0.62 ***^,##^
Maximum pain	0.59 ***^,###^	0.38 *	0.48 ***^,#^	0.26 *	0.56 **^,#^
PDI	0.03	0.12	0.47 **	0.15	0.32
Negative emotions	0.11	−0.08	0.36 *	0.07	0.24
Sense of threat	−0.08	0.15	0.48 **	0.17	0.22
Anxiety	0.31 *	X	X	0.27 *	0.39 *
Depression	0.24	X	X	0.19	0.44 *
Anger	−0.14	X	X	−0.13	−0.24
PTSD	0.00	X	X	0.22	0.01
Intrusion	0.08	X	X	0.21	−0.03
Arousal	0.01	X	X	0.28 *	0.12
Avoidance	−0.14	X	X	0.09	−0.03

Statistical significance: * *p* < 0.05, ** *p* < 0.01, *** *p* < 0.001. Statistical significance after Bonferroni correction: ^#^ *p* < 0.05, ^##^ *p* < 0.01, ^###^ *p* < 0.001.

**Table 7:** Correlations between the intensity of average pain and selected demographic, medical, and psychological variables (Spearman’s rho).

	Controls	ICU	NICU	Oncology	Observation
Age	0.21	0.14	−0.02	0.09	−0.02
Age of youngest child	0.24	0.36	−0.34	0.04	−0.22
BMI	0.03	−0.05	0.34 *	0.04	−0.32
No. of pregnancies	0.12	−0.04	0.04	0.03	−0.24
No. of births	0.10	−0.06	0.06	0.11	−0.30
Social support	−0.21	−0.33	−0.10	−0.26	−0.19
Maximum pain	0.71 ***^,###^	0.83 ***^,###^	0.82 ***^,###^	0.80 ***^,###^	0.90 ***^,###^
PDI	0.06	−0.05	0.02	0.05	0.05
Negative emotions	0.06	0.12	0.08	−0.21	−0.04
Sense of threat	0.06	−0.24	0.09	0.20	0.17
Anxiety	0.45 ***^,#^	X	X	0.09	0.27
Depression	0.37 **	X	X	−0.03	0.21
Anger	−0.24	X	X	0.01	−0.41 *
PTSD	0.02	X	X	−0.14	−0.14
Intrusion	0.02	X	X	−0.20	−0.01
Arousal	0.01	X	X	−0.14	−0.07
Avoidance	0.02	X	X	−0.12	−0.44 *

Statistical significance: * *p* < 0.05, ** *p* < 0.01, *** *p* < 0.001. Statistical significance after Bonferroni correction: ^#^ *p* < 0.05, ^###^ *p* < 0.001.

**Table 8:** Correlations between the intensity of maximum pain and selected demographic, medical, and psychological variables (Spearman’s rho).

	Controls	ICU	NICU	Oncology	Observation
Age	0.17	−0.08	−0.01	0.04	0.13
Age of youngest child	0.26	0.09	−0.41 *	0.12	−0.08
BMI	0.12	−0.04	0.37 *	−0.08	−0.33
No. of pregnancies	0.16	−0.11	0.15	−0.02	−0.19
No. of births	0.12	−0.15	0.18	0.06	−0.25
Social support	−0.19	−0.36	−0.30	−0.18	−0.03
Average pain	0.71 ***^,###^	0.83 ***^,###^	0.82 ***^,###^	0.80 ***^,###^	0.90 ***^,###^
PDI	0.03	0.15	0.17	0.12	0.01
Negative emotions	0.08	0.19	0.12	−0.13	−0.09
Sense of threat	0.01	−0.02	0.23	0.22	0.13
Anxiety	0.40 **^,#^	X	X	0.20	0.21
Depression	0.37 **	X	X	0.12	0.17
Anger	−0.31 *	X	X	−0.02	−0.53 **^,#^
PTSD	0.05	X	X	−0.02	−0.15
Intrusion	0.05	X	X	−0.04	−0.09
Arousal	0.07	X	X	−0.02	−0.02
Avoidance	−0.02	X	X	−0.07	−0.44 *

Statistical significance: * *p* < 0.05, ** *p* < 0.01, *** *p* < 0.001. Statistical significance after Bonferroni correction: ^#^ *p* < 0.05, ^###^ *p* < 0.001.

## Data Availability

Data are available in the Department of Anaesthesiological and Intensive Care Nursing, Faculty of Health Sciences, Medical University of Lublin, Chodzki Street 7, 20-093 Lublin, Poland.
